# VACCIMEL, an allogeneic melanoma vaccine, efficiently triggers T cell immune responses against neoantigens and alloantigens, as well as against tumor-associated antigens

**DOI:** 10.3389/fimmu.2024.1496204

**Published:** 2025-01-07

**Authors:** Ibel Carri, Erika Schwab, Juan Carlos Trivino, Erika M. von Euw, Morten Nielsen, José Mordoh, María Marcela Barrio

**Affiliations:** ^1^ Centro de Investigaciones Oncológicas (FUCA), Fundación Cáncer, Ciudad Autónoma de Buenos Aires, Argentina; ^2^ Instituto de Investigaciones Biotecnológicas, Universidad Nacional de San Martín (UNSAM) – Consejo Nacional de Investigaciones Científicas y Técnicas (CONICET), Buenos Aires, Argentina; ^3^ Bioinformatic Department, Sistemas Genómicos, Valencia, Spain; ^4^ Translational Oncology Research Labs, Jonsson Comprehensive Cancer Center, University of California, Los Angeles, Los Angeles, CA, United States; ^5^ Department of Health Technology, Technical University of Denmark, Lyngby, Denmark

**Keywords:** cancer immunotherapy, whole cancer cell vaccine, allogeneic vaccine, VACCIMEL, neoantigen, melanoma

## Abstract

VACCIMEL is a therapeutic cancer vaccine composed of four irradiated allogeneic human melanoma cell lines rationally selected to cover a wide range of melanoma tumor-associated antigens (TAA). We previously demonstrated that vaccination in the adjuvant setting prolonged the distant-metastasis-free survival of cutaneous melanoma patients and that T cells reactive to TAA and the patient’s private neoantigens increased during treatment. However, immune responses directed to vaccine antigens that may arise from VACCIMEL’s somatic mutations and human polymorphisms remain unexplored. To study these immunogens, we performed whole-exome sequencing of paired tumor and germinal samples from four vaccinated patients and the vaccine cells. VACCIMEL variants were called by comparing the vaccine and the patient’s exomes, and non-synonymous coding variants were used to predict T cell epitopes. Candidates were ranked based on their mRNA expression in VACCIMEL, predicted peptide-HLA (pHLA) presentation, and pHLA stability. Then, the immune responses to prioritized epitope candidates were tested using IFNγ ELISpot assays on vaccinated patients’ PBMC samples. The comparison of the vaccine with the patients’ germinal exomes revealed on average 9481 coding non-synonymous variants, suggesting that VACCIMEL offers a high number of potential antigens. Between 0,05 and 0,2% of these variants were also found in the tumors of three vaccinated patients; however, one patient with a high tumor mutational burden (TMB) shared 19,5% somatic variants. The assessment of T cell responses showed that vaccinated patients mounted highly diverse responses against VACCIMEL peptides. Notably, effector T cells targeting the patient’s tumor antigens, comprising neoantigens and TAA, were found in higher frequencies than T cells targeting VACCIMEL-exclusive antigens. On the other hand, we observed that the immunogenic epitopes are not conserved across patients, despite sharing HLA and that immune responses fluctuate over time. Finally, a positive correlation between VACCIMEL antigen expression and the intensity of the T cell responses was found. Our results demonstrate that the immune system simultaneously responds to a high number of antigens, either vaccinal or private, proving that immune responses against epitopes not expressed in the patient’s tumors were not detrimental to the immune recognition of neoantigens and TAA.

## 1 Introduction

During the last two decades, the role of the antitumoral immune response in tumor control has been evidenced, and many immunotherapeutic approaches have been evaluated in clinical trials with encouraging results in multiple cancer types (1). Among these, whole-tumor cell vaccines arise as an option that provides a wide range of antigens to re-educate the immune system toward tumor recognition and elimination. These vaccines can be produced from autologous or allogeneic tumor samples or cell lines, combined with adjuvants such as granulocyte and macrophage colony-stimulating factor (GM-CSF) and Bacillus Calmette-Guérin (BCG). Autologous vaccines would be preferable regarding the anticancer immune response because they contain all the patient’s specific tumor antigens (2). However, their preparation process is laborious and difficult to systematize; besides, tumor samples are not always available. In this scenario, allogeneic whole-tumor cell vaccines appear as good alternatives. They are a generic source of shared tumor-associated antigens (TAA), which are either tissue-specific antigens, tumor-enriched proteins, or development-specific antigens (i.e. oncofetal, cancer-testis) (3). Moreover, allogeneic cells can be manufactured beforehand, being quickly available for their use off-the-shelf (4). Yet, the effectiveness of this strategy is subjected to a proper selection of cell lines that provide enough and diverse antigens covering the highly heterogeneous human tumors (5).

A concern regarding allogeneic whole-tumor vaccines is the non-tumor-specific immune response stimulated by alloantigens (6). It is believed that the allo response may be directed against the Human Leukocyte Antigens (HLA) (7). Still, a variable number of other alloantigens can arise from multiple polymorphisms, depending on the germinal background of the recipient patients (8). It is not clear to what extent these allogeneic molecules might capture the attention of the immune system, acting like enhancers and/or competitors with relevant tumor antigens, such as TAA and neoantigens. Despite this argument, there is evidence of allogeneic components acting as enhancers of antitumoral immune responses (5, 9).

Besides TAA and alloantigens, whole-tumor cell vaccines carry somatic mutations that generate tumor-specific antigens (TSA) or neoantigens. The neoantigen fraction expected to have a direct antitumor effect in vaccinated patients would be that also expressed in their tumors. These are called public neoantigens, and their therapeutic benefits have been largely discussed (10, 11). First, they are good targets for off-the-shelf immunotherapy (10). Also, they are more likely to be located in driver genes that due to their relevance in tumor biology, tend to be clonally conserved across metastases, making them ideal tumor-specific molecules that target the whole neoplastic mass (12). However, taking into account the large extension of the human genome, it seems unlikely that random variants, and their derived TSA, are shared across tumors. Another factor that decreases the likelihood of generating public neoepitopes is the high polymorphism of HLA molecules that restrict the neoepitopes. In consequence, most identified neoantigens are private (13, 14) although an enrichment of neoantigens from driver mutations has been observed in some tumors (11, 15). Furthermore, Mac Keon et al. (16) demonstrated in a murine model that vaccination with dendritic cells (DCs) loaded with the syngeneic B16F1 cell line or allogeneic Cloudman irradiated cells was more effective when the tumor cells contain neoantigens in addition to TAA. Therefore, it is relevant to study “how public” are the neoantigens present in whole-tumor allogeneic cellular vaccines, and to what extent they can contribute to the antitumoral effect observed in responder patients.

VACCIMEL is a therapeutic cancer vaccine recently approved in Argentina that demonstrated a significant benefit in distant metastasis-free survival (DMFS) of cutaneous melanoma patients treated in adjuvancy in a phase II study (17, 18). This vaccine is composed of four lethally irradiated allogeneic human cell lines derived from melanoma patients (Mel-XY1, Mel-XY2, Mel-XY3, and Mel-XX4), that were rationally selected to cover a wide range of melanoma TAA. VACCIMEL is administered with BCG and rhGM-CSM, two potent proinflammatory adjuvants (19, 20). Immune responses against abundant TAA present in VACCIMEL were demonstrated in vaccinated patients. Also, immune responses against private neoantigens were shown in patients after receiving VACCIMEL. Importantly, it was observed that the frequency of T cells reactive to both kinds of antigens increased during the two-year treatment. Thus, vaccination may have induced antigen spreading and/or a strong immunomodulatory effect towards tumor recognition, broadening the immunotherapeutic effect of VACCIMEL (21, 22). However, other antigens that can be generated through human polymorphisms and somatic mutations of VACCIMEL are still unexplored.

In this work, we studied four melanoma patients treated with VACCIMEL and analyzed their exomes as well as their T cell immune responses against multiple VACCIMEL’s TAA, TSA, and alloantigens. Through this study, we delineate answers to the following questions i) What kind/types of antigens does VACCIMEL immunize against? ii) how many antigens can VACCIMEL simultaneously immunize to? and iii) what are the characteristics of immunogenic peptides in cellular vaccines like VACCIMEL? Further, considering the intrinsic patient variability and tumor heterogeneity (23), we inquired iv) if the antigenic landscape is conserved across multiple melanoma patients by analyzing neoepitope data from another cohort.

## 2 Results

### 2.1 Patients clinical status

To approach the questions raised in the introduction, we selected four cutaneous melanoma patients from the Phase II CASVAC 0401 study who were randomized to the VACCIMEL arm and completed the vaccination protocol consisting of 13 doses during a two-year period. These patients had a good outcome to the treatment (DMFS > 24 months) (24). The initial clinical stages of the selected patients, as well as the DMFS and overall survival (OS) are shown in **Table 1**.

**Table 1:** Evolution of studied patients until April 2024.

Patient	Sex	Age*	Stage (AJCC)	DMFS	OS	Citation
#005	F	33	IIIC	166	166+	(17, 18, 25)
#006	F	51	IIIB	25	79	(17, 18)
#032	F	43	IIIB	121+	121+	(17, 18)
#045	M	41	IIIA	57	95+	(25, 26)

*Age at the time of inclusion in the VACCIMEL clinical trial. DMFS, Distant metastases-free survival; OS, Overall survival, +, Ongoing. DMFS and OS are reported in months.

The basis for the selection of these patients was that tumor samples to perform whole-exome sequencing (WES) were available and that they all had positive IFNγ enzyme-linked immunospot (ELISpot) responses when post-vaccination peripheral blood mononuclear cells (PBMC) samples were stimulated with the vaccine lysate (18, 20, 26). In the next section, we will dissect this immune response to identify the specificities of the antigens that triggered it.

### 2.2 Types of antigens contained in VACCIMEL

**Figure 1:**
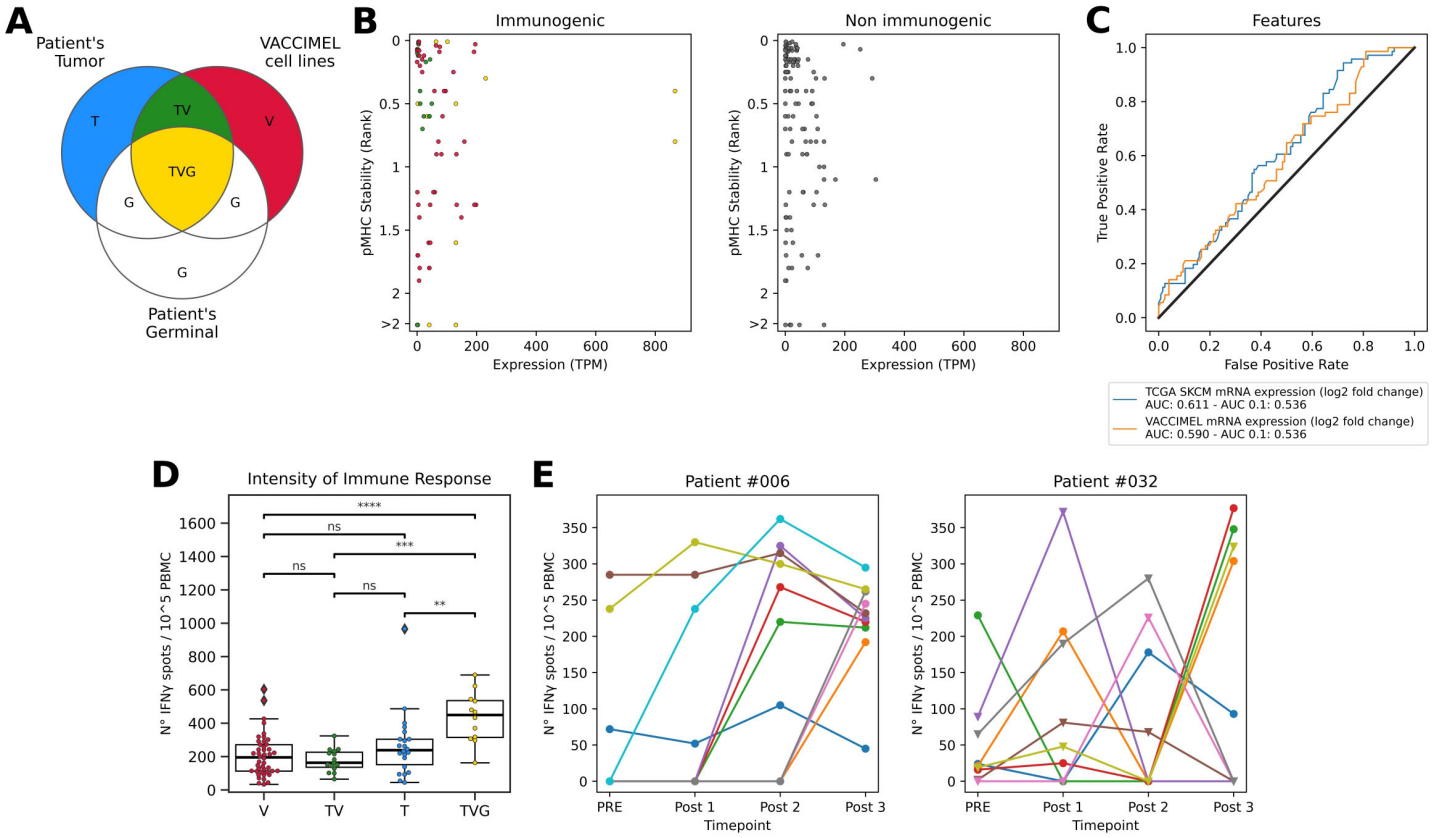
**(A)** Venn diagram of molecules presented to the immune system of vaccinated patients. T: Patient’s tumor private neopeptides (blue); V: VACCIMEL molecules derived from polymorphisms and somatic mutations not present in each of the vaccinated patient’s germline or tumor (red); G: Patient’s germinal molecules (white); TV: Public neopeptides shared between patient’s tumor and VACCIMEL (green); TVG: Germinal non-mutated molecules present in the patient’s tumor and VACCIMEL, including TAA (yellow). **(B)** Features of peptides tested for T cell responses. Left: Immunogenic peptides colored according to their source. Right: Non-immunogenic peptides. In the y-axes, pHLA stability is reported as %Rank score, with lower values representing top-ranked peptides. In the x-axes, the expression values in TPM correspond to the sum of the expression in the four cell lines rescaled to one million. **(C)** Immunogenicity predictive performance of the top peptide features according to AUC and AUC 0.1. **(D)** Intensity of T cell responses evaluated as reactive T cell frequency in ELISpot assay per antigen category. V: epitopes exclusive from the vaccine. TV: shared neoepitopes expressed both in tumor and vaccine. T: private neoepitopes previously tested in (22). TVG: TAA expressed both in the vaccine, tumor, and germinal, previously tested in (21). The statistics were calculated using the Mann-Whitney-Wilcoxon test. * p < 0.05; ** p < 0.01; *** p < 0.001; **** p < 0.0001; ns, non-significant. **(E)** Neoantigens from patients #006 (left) and #032 (right) tested in multiple time points. PRE: before immunization, Post 1: 6 months after starting the vaccination protocol, Post 2: 12 months after starting the vaccination protocol, and Post 3: 25 months after starting the vaccination protocol and one month after receiving the last VACCIMEL dose. The shape of the dot represents the peptide category. Triangle: shared neopeptides expressed both in the tumor and the vaccine or category VT. Circle: private neopeptides previously tested in (22) or category T. ELISpot counts correspond to the average number of triplicate spots observed on peptide-stimulated effector cells triplicates minus the spots observed in unstimulated effector cells (cultured with non-pulsed APC), relative to 100.000 effector cells.

To study the sources of antigens offered by the whole-tumor cell vaccine VACCIMEL, it is pertinent to establish and investigate the immune properties of the categories outlined in **Figure 1A**. TAA expressed in the vaccine cells are non-mutated immunogenic molecules that are shared between the patient’s tumor, the vaccine, and the germinal tissue (TVG). In addition, VACCIMEL expresses molecules that do not overlap with the patient’s self and are therefore likely to produce an immune response. These comprise the molecules derived from human polymorphisms and somatic mutations of the VACCIMEL component melanoma cell lines. The former will be off-target antigens (V), and the latter could include shared neoantigens (TV). Also, the immunotherapy might induce the immune recognition of the patient’s tumor private neoantigens (T) that are not expressed in VACCIMEL’s cell lines.

We have previously demonstrated that PBMC of vaccinated patients are reactive against selected TAA-derived peptides, comprising melanocytic differentiation antigens and cancer testis antigens; showing a progressive increase in the frequency of specific T cells throughout the 2-year vaccination protocol (18, 21). Similarly, we showed that patients developed T cell responses targeting private neoantigens along the VACCIMEL’s vaccination protocol (21, 22). To further study antigens derived from VACCIMEL genomic variants (V and TV), we performed WES of VACCIMEL cell lines and germinal samples from the four vaccinated patients included in this study. Then, we proceeded to identify potentially immunogenic variants by comparing the germinal tissue of each patient to VACCIMEL cell lines’ exomes, as well as the patient’s tumors. We limited the analysis to the identification of single nucleotide variants (SNVs) and short insertions and deletions (InDels). Then, we inspected non-synonymous coding variants with a variant allele frequency greater than 10%, from expressed genes according to RNAseq (transcripts per million (TPM) > 0) (see Materials and Methods). This analysis revealed 9481 ± 270 (average ± standard deviation) VACCIMEL-specific variants (i.e. variants different from the patients’ germinal DNA), and this number of VACCIMEL variants is conserved to a high degree across the four vaccinated patients (**Table 2**, columns V plus TV).

**Table 2:** Variant counts per patient.

	Variants		
Patient	Vaccine only (V)	Vaccine and Tumor (TV)	Tumor only (T)
#005	9540	5	219
#006	9074	9	153
#032	7770	1882	1591
#045	9623	21	644

Exomic variants that could generate antigens by being non-self were studied by pairing the patients’ germline exome with that of the VACCIMEL cell lines and with their tumors. V: Variants only found in VACCIMEL cell lines, TV: Variants found both in VACCIMEL cell lines and the patient’s tumors, and T: Variants only found in the patient’s tumors.

To identify how many of these variants are cancer-related, we used the VACCIMEL cell lines’ exomes to identify somatic mutations using MuTect2 in tumor-only mode. Then, we intersected these mutations with the variants obtained by comparing the patient’s germinal tissue with VACCIMEL. This analysis revealed that at least 13-15% of the variants were somatic mutations. By analyzing the mutational signatures of the four VACCIMEL cell lines, we confirmed that all of them preserve mutational profiles of UV exposure, which are linked to melanoma development (**Supplementary Table S1**, **Supplementary Figure S1**). SBS5, resulted to be the predominant signature. These mutations are likely due to residual germline variants that persist when somatic mutations are identified in absence of matching normal samples (27).

By inspecting the somatic variants of the patient’s tumors, a great variability between patients was found, both in terms of their tumor mutational burden (TMB) (**Supplementary Table S2**) and the overlap with VACCIMEL variants (**Table 2**, column TV). Of note, only patient #032 displayed a very high TMB, and most of these patient tumor’s variants were shared with the vaccine. Except for this patient, our results overall indicate that in general there is low overlap between VACCIMEL’s and the patient’s tumor variants.

The high number of variants identified in VACCIMEL suggests that the vaccine might provide an enormous load of potential antigens. To investigate this, we predicted T cell epitope candidates (**Table 3**) as peptides derived from the identified variants with a high likelihood of presentation in the patient’s HLA class I molecules (**Supplementary Table S3**) according to NetMHCpan 4.1 (%Rank_EL < 0.5) (28). Notably, HLA restriction drastically reduced the number of candidate epitopes compared to the number of variants. In this sense, it was interesting to study patient #045, who displays similar numbers of predicted tumor antigens (comprising T and TV) [41] to that of patients #005 [21] and #006 [40] even when having a higher TMB (**Supplementary Table S2**). This can be explained, at least in part, by the homozygosity of all HLA class I loci for this patient, highlighting the interplay between the TMB and the HLA haplotype (29). Still, neoepitope candidates from patient #032 are strikingly higher than the number of neoepitope candidates from the other patients.

**Table 3:** Candidate epitope counts per patient.

	Candidate Epitopes		
Patient	Vaccine only (V)	Vaccine and Tumor (TV)	Tumor only (T)
#005	210	1	20
#006	173	5	35
#032	156	766	130
#045	129	4	37

Peptides derived from variants listed in **Table 1** which are predicted to bind to the corresponding patient’s HLA. V: Peptides from variants only found in VACCIMEL cell lines, TV: Peptides from variants found both in VACCIMEL cell lines and the patient’s tumors, T: Peptides from variants only found in the patient’s tumors.

Next, we made a stringent selection of epitope candidates to test for T cell responses. The peptides were selected considering a predicted high likelihood of HLA antigen presentation and other criteria such as predicted peptide-HLA (pHLA) complex stability and transcript abundance as determined by RNAseq (see Materials and Methods). For each patient, the final selection included peptides exclusive from the vaccine (V). Only for patient #032 we included shared neoantigens (TV), because TV peptides from the other patients did not meet the filtering criteria. Also, to guarantee that the T cell responses observed in the ELISpot are triggered by cytotoxic CD8+ T cells, strong binding of the candidates to the patient’s HLA class II molecules was discarded using NetMHCIIpan 4.3 (30) (%Rank_EL < 1). IFNγ ELISpot assays were performed using available post-vaccination PBMC samples from patients #006, #032, and #045 (ELISpot example pictures in **Supplementary Figure S2**). The PBMC samples were obtained 25 months after starting the vaccination protocol and one month after receiving the last dose. Of note, in previous studies we have tested reactivity to TAA and VACCIMEL lysate making these samples scarce, and limiting the number of candidates to be tested. In fact, we could not test any VACCIMEL-exclusive candidates for patient #005 due to the lack of remaining PBMC.

Reactive T cells were found in all tested categories (**Table 4**, **Supplementary Table S4**), validating that VACCIMEL stimulates immune responses against both alloantigens and neoantigens, including shared neoantigens as evidenced for patient #032, since immune responses to VACCIMEL were absent before vaccination (18). Additionally, the immune response stimulated by the vaccination was not circumscribed to antigens expressed in VACCIMEL cell lines, as we have previously observed post-vaccination T cell responses against the patient’s private neoantigens, which are shown in the last column of **Table 4** (21, 22).

**Table 4:** Vaccine and tumor tested epitopes and percentage of positivity per patient.

	Tested Epitopes		
Patient	Vaccine only (V) (positives/tested)	Vaccine and Tumor (TV) (positives/tested)	Tumor only (T) (positives/tested)
#005	NA	NA	7/21 (33,3%)
#006	2/46 (4,3%)	NA	10/47 (21,3%)
#032	16/43 (37,2%)	15/46 (32,6%)	5/12 (41,7%)
#045	26/47 (55,3%)	NA	NA

These peptides are derived from the epitope candidates listed in **Table 2**. V: Peptides from variants only found in VACCIMEL. TV: Peptides from variants found both in VACCIMEL and the patient’s tumors. T: Peptides from variants only found in the patient’s tumors, studied in Carri et al. (22). Of note, these peptides include predicted weak HLA binders. ELISpot was performed in triplicate and positivity was determined as described under methods. NA, not assessed.

Interestingly, some identified neoepitopes originated in mutations of cancer driver genes (31). Two neoepitopes generated in variants found only in the vaccine (**Figure 1A**, category V) derived from mutations in the kinetochore localized astrin binding protein (KNSTRN) and mitogen-activated protein kinase 1 (MAP2K1) oncogenes. Two private neoepitopes (**Figure 1A**, category T) derived from mutations in the FAT atypical cadherin 4 (FAT4) and Rho GTPase activating protein 26 (ARHGAP26) tumor suppressor genes. Additionally, four VACCIMEL neoepitopes originated in mutations observed earlier in other melanomas. The neoepitopes I ***S*** SKFKSKR (DDX47) and IEIEDTFETL ***W*** (TGFBI) corresponded to category V (**Figure 1A**), and HSTLQKSL ***W*** (LRR1) together with KLRKKQNE ***R*** (SLFN12) neoepitopes corresponded to category TV (**Figure 1A**), being expressed both in VACCIMEL and patient #032’s tumor. Finally, 29 neoepitopes from category V and 12 shared neoepitopes from category TV derive from cancer somatic mutations curated in COSMIC (32).

Considering that HLA molecules are highly immunogenic alloantigens that are abundantly expressed in VACCIMEL (**Supplementary Table S5**) we also inspected VACCIMEL’s epitope candidates from this source. The pipeline implemented to identify variants in VACCIMEL comprises the alignment to the human reference genome and the comparison of exomes with MuTect2, which is not optimal to find mismatches in the HLA locus due to its high variability. Therefore, we performed VACCIMEL HLA genotyping with Optitype (33) and retrieved the reference HLA allelic sequences from IPD-IMGT/HLA (34), as described under methods. Following, we compared VACCIMEL’s HLA with the patients’ to derive epitope candidates. This analysis revealed between 23 and 64 HLA’s epitope candidates presented in class I molecules per patient (**Supplementary Table S6**). Since HLA alloantigens stimulate strong B cell immunity which is sustained by CD4 T cells, we also studied epitope candidates presented in the context of the patients’ HLA class II molecules, obtaining between 63 and 168 candidates per patient. It should be mentioned that T cell immune responses against HLA epitope candidates have not been experimentally tested.

Altogether these results evidence that the immune response stimulated by VACCIMEL is highly diverse and that immune responses against off-target antigens do not impede the relevant immune responses against TAA and TSA already demonstrated for the vaccinated patients. In addition, the identification of neoepitopes from driver and frequent mutations in cancer suggests that VACCIMEL might offer TSA besides TAA, as demonstrated for patient #032.

### 2.3 Characteristics of immunogenic peptides from VACCIMEL and patient’s private neoantigens

Because not all the potentially immunogenic VACCIMEL epitope candidates (**Figure 1A**, categories V, TV, and TVG) triggered T cell responses, we inquired which characteristics would define their immunogenicity. Considering that after inoculation to the patients, the vaccine cells enter a process of degradation and loading in HLA molecules from antigen-presenting cells (APCs) (35), we hypothesized that i) peptides from highly abundant proteins, ii) peptides with a high likelihood of HLA antigen presentation, and iii) peptides that form very stable pHLA complexes would have more chances to be loaded onto HLA molecules, and be presented for a longer time, increasing their odds of T cell recognition.

To challenge this hypothesis, in **Figure 1B** we plotted the dispersion in pHLA stability and mRNA abundance in VACCIMEL cells, as determined by RNAseq for the positive and negative peptide subsets. In this way, we covered all ranges (high, medium, low) for these two latter features, screening peptides that represented all the possible values of the range (**Supplementary Table S4**). For this analysis, we also included immune responses targeting TAA expressed in VACCIMEL (**Figure 1A**, category TVG) that were previously tested in the same patients (21). While we found a correlation between the gene expression in VACCIMEL and the development of an immune response, r (196) = 0.178, p = 0.01, there was no clear association between the immune responses and pHLA stability.

Next, we explored additional features that might shed light on the rules of immunogenicity of VACCIMEL epitopes. Peptide binding to the HLA can be a confounding variable when evaluating the performance of immunogenicity predictive methods, because it is a prerequisite for triggering a T cell response. Therefore, this analysis was limited to peptides that were predicted as strong HLA binders. We included features describing the mutation type, the antigen expression, the antigen likelihood of processing and presentation, the peptide self-similarity or similarity to known antigens, the likelihood of T cell recognition, and the peptide physicochemical properties. The definition of the features and the details for their calculation are detailed under Materials and Methods. Next, we evaluated the classification performance of these features in terms of the area under the curve of the receiver operating characteristic (AUC) and the partial AUC at 0.1 (AUC 0.1), which best reflects the specificity (**Figure 1C**, **Supplementary Figure S3**, **Supplementary Table S7**). Our results shown in **Figure 1C** suggest that the features that would favor the immunogenicity of the candidate peptides are related to gene expression in the vaccine.

Considering that highly immunogenic antigens stimulate strong immune responses, we explored the immunogenic potential of the identified epitopes by comparing the number of IFNγ spots in the ELISpot assays (**Figure 1D**, **Supplementary Figure S4**). This analysis revealed that T cell responses against TAA (TVG) are the most prevalent (p < 0.0001), followed by T cells targeting private neoantigens (T).

We also studied the correlation between the number of IFNγ spots generated by immunogenic epitopes and the calculated features. A correlation between mRNA expression in VACCIMEL and the number of IFNγ spots was found, r(196) = 0.22, p < 0.001. In a consistent manner, shared neoepitopes (TV) stimulated the lower number of IFNγ spots and were also the least expressed among all tested peptides (**Supplementary Figure S5**).

To assess the immunoprevalence of an antigen, it is necessary to study a group of immunized subjects that enable the calculation of the response frequency. Usually, this is not explored in cancer patients because most tumor antigens are private, since they are derived from random somatic mutations. However, patients #006 and #032 shared the same two HLA-B alleles (**Supplementary Table S3**), so we had the opportunity to test 18 VACCIMEL candidate epitopes in both patients. We found concordant immune responses for 8 candidate epitopes (44%) of which 3 were immunogenic and 5 were non-immunogenic in both patients. Importantly, the remaining 10 candidates were only immunogenic in one or another patient (**Table 5**). In light of this observation, it appears that patient-specific characteristics and/or immune stochasticity influence vaccine-directed immunity.

**Table 5:** Peptides tested both in patients #006 and #032.

Antigen	Epitope	HLA	#006 immune response	#032 immune response	Category
PLA2G4B	LPSKDLVI	HLA-B5101	–	–	V
NPHP4	KAVSATEPVTF	HLA-B5701	–	–	V
TBC1D1	LIKEDAVHW	HLA-B5701	–	–	TV
HRCT1	RAQPWPFRW	HLA-B5701	–	–	V
INSR	RSYALVSLF	HLA-B5701	–	–	V
ZDHHC12	NPFDRGLTRI	HLA-B5101	+	+	TV
KCTD17	KTKLLQARGTW	HLA-B5701	+	+	V
MAGEC1	FPSSTSSI	HLA-B5101	–	+	V
MARCH7	RPKENSMSI	HLA-B5101	–	+	V
MRPL38	IGLPPPKVSW	HLA-B5701	–	+	V
TTC37	KSIDLYLAL	HLA-B5701	–	+	V
NUB1	KTNGGRCRIW	HLA-B5701	–	+	V
KLHL30	RSLLALSSPYF	HLA-B5701	–	+	V
DNMT3B	TSWPSPPSSY	HLA-B5701	–	+	V
L3MBTL2	FPSYNSSV	HLA-B5101	+	–	V
CREBBP	LPAQPQPSPV	HLA-B5101	+	–	V
HSPG2	VPLSPATNMSV	HLA-B5101	+	–	V

In Podaza et al. (21), the vaccinated patients’ immune responses against TAA were measured during the vaccination protocol, showing a progressive increase in most cases. Therefore, in this study we tested candidate epitopes in PBMC samples obtained at the end of the vaccination protocol (hereinafter referred to as post 3), where we consider it most likely to find immunogenic epitopes. However, some tumor neoantigens were evaluated in patients’ PBMC samples obtained at multiple time points during the vaccination protocol (21, 22) (**Figure 1E**, **Table 6**). In particular, these results disclose the absence of several immunogenicity readouts in the post 3 sample for peptides demonstrated to be immunogenic in post 1 and/or post 2 samples, obtained at 6 and 12 months after starting to receive the vaccine, respectively. This observation further underlines the challenge of accurately defining the peptide’s immunogenicity status using a single measurement.

**Table 6:** Neoantigens from patients #006 and #032 tested in more than one time point.

Patient	Category	Antigen	Neoepitope	PRE	Post 1	Post 2	Post 3
#006	T	ZNF416	KSKLVRHQRI	72	52	105	45
#006	T	B4GALNT3	PVKNLPQV	0	0	0	192
#006	T	IPO8	NISEDVIFF	0	0	220	212
#006	T	PTPRU	DHYAYSYYL	0	0	268	220
#006	T	PRICKLE3	KRIPLPPHLC	0	0	325	225
#006	T	TRIOBP	RTSSTQQDNPK	285	285	315	232
#006	T	IFT80	DDIYPIDFY	0	0	0	245
#006	T	PRICKLE3	SSEDDGFFLGK	0	0	0	262
#006	T	ZNF878	ALSYLVSFQR	238	330	300	265
#006	T	IQCF2	LVRRTLLHV	0	238	362	295
#032	TV	ITGAL	STALRLTAF	89	372	0	0
#032	TV	PCNT	TSLPQTQGL	2	81	68	0
#032	TV	ALDH3B2	VTLGHGLPELY	0	0	226	0
#032	TV	CD177	ALAPALWWR	65	190	280	0
#032	T	LHB	AWASREPLRPR	24	0	178	93
#032	T	PPP1R15A	QSAHFRGW	229	0	0	304
#032	TV	CRYBB3	SPDHKLHLF	19	48	0	324
#032	T	ADM2	AALGCISLLW	16	25	0	348
#032	T	KRT40	SSQLAQIQR	21	207	0	377

ELISpot counts correspond to the average number of spots observed on peptide-stimulated effector cells triplicates minus the spots observed in unstimulated effector cells (cultured with non-pulsed APC), relative to 100.000 effector cells. The grayscale of the cells in the table reflect the intensity of the immune responses.

### 2.4 Study of neoantigens shared between VACCIMEL and other melanoma patients

**Figure 2:**
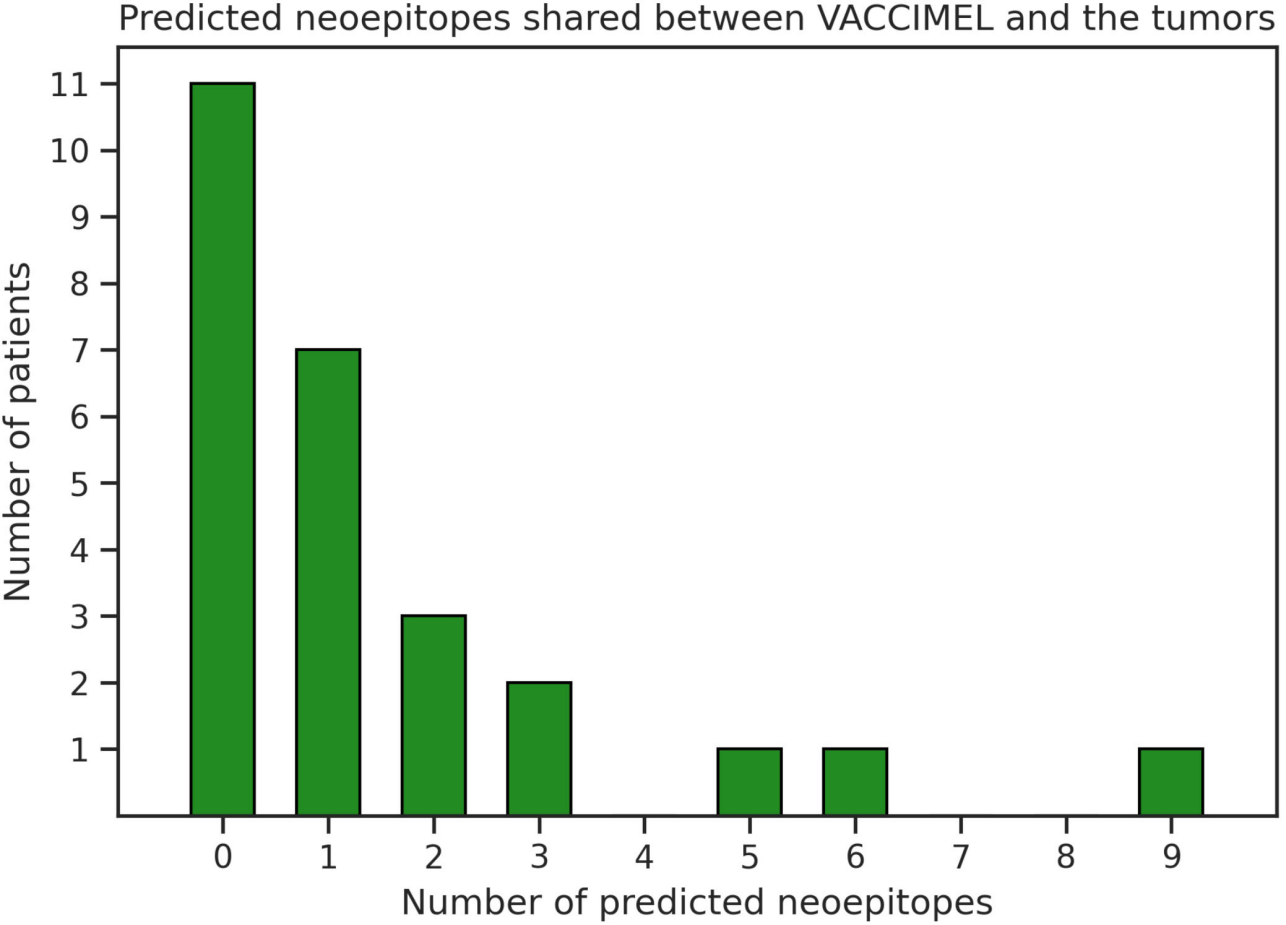
Number of predicted neoepitopes shared between VACCIMEL and melanoma tumors from another cohort (15, 36).

We observed that one (patient #032) out of four vaccinated patients analyzed here presented a high number of neoepitopes shared with VACCIMEL (category TV). Consequently, we wanted to investigate how frequently melanoma tumors present an overlap with VACCIMEL’s neoantigens. To this aim, we analyzed an independent cohort of cutaneous melanoma patients (n = 26) treated with adoptive cell transfer of tumor-infiltrating lymphocytes (TILs) in which immunity against neoantigens was studied (15, 36). We inspected the intersection between the patient’s neoepitopes and peptide sequences derived from VACCIMEL’s somatic mutations that were identified using MuPeXI (37). Of the 26 patients, 15 (58%) have at least one predicted neoepitope (%Rank_EL < 2) shared with VACCIMEL, and 9 (35%) have more than one candidate (**Figure 2**, **Supplementary Table S8**). Additionally, a correlation between the patient’s TMB and the number of shared predicted neoantigens was found; r(24) = 0.49, p = 0.01.

In the original publications (15, 36), the authors evaluated if the melanoma patients developed T cells targeting some selected neoepitope candidates by using barcode-labeled pHLA multimers with TIL samples. This experimental dataset included 8 candidate neoepitopes shared with VACCIMEL (category TV), of which only the neopeptide GLQEERVQL (RP1L1) was immunogenic.

Finally, we also searched in CEDAR (38) and NEPdb (39) public databases for experimentally validated immune responses of melanoma patients against neoepitopes derived from VACCIMEL’s somatic mutations. Only the neoepitope LAT ***E*** KSRWS (BRAF V600E) restricted to the HLA-A02:01 was found in CEDAR reported as immunogenic in two patients (40). Thus, there is limited evidence of immune responses against these antigens (TV).

## 3 Discussion

A general concern regarding allogeneic vaccination is to understand to what extent human polymorphisms are a source of alloantigens, and if they could interfere with triggered cancer-specific immune responses. In this work, we have studied the immunogenic potential of different types of antigens in four melanoma patients treated with the whole-tumor allogeneic vaccine VACCIMEL to answer these questions. We have previously shown that VACCIMEL triggers strong immune responses against TAA as well as towards neoantigens (21). Here, in a more exhaustive study, we demonstrate that VACCIMEL can stimulate immune responses to the patients’ private neoantigens, as well as to VACCIMEL neoantigens and alloantigens, suggesting that the human immune system is capable of simultaneously addressing a large number of antigens. Considering that here we only validated the best-ranked HLA class I restricted epitope candidates, and that we have not evaluated antigens derived from epigenetic changes or post-transcriptional/post-translational modifications, which are additional potential sources of possible shared immunogenic neoepitopes (41–45), we could infer that VACCIMEL stimulates an immune response towards a great number of epitopes, probably more than the ones identified in this limited experimental set.

To explain in which manner VACCIMEL can trigger such reactivity, it should be considered that the vaccination protocol encompasses several vaccination doses [13] over two years of treatment. Repeated VACCIMEL plus BCG and GM-CSF vaccination progressively fosters patient’s immunity, as revealed by increasing delayed-type hypersensitivity (DTH) in responding patients (18). We would like to propose the following hypothesis: during the first vaccinations, stronger immunogens such as TAA, trigger a first wave of cytotoxic T lymphocytes that destroy some occult micrometastasis, releasing more TAA, as well as private neoantigens. These newly released antigens would be captured and processed by APC, determining a new wave of effector T cells, now also aiming at private neoantigens. This hypothesis is sustained by the fact that the response to several private neoantigens is increased towards the end of the vaccination protocol. *Ab initio* we only found weak T cell responses targeting a few private neoantigens, and notably these responses increased with vaccination (21). This hypothesis also provides a possible explanation for the highly and progressively augmented immune responses against shared TAA. Considering that TAA are also expressed in the patient’s tumors, the immune-mediated destruction of the micrometastasis might also serve as a reboost for TAA-directed T cells, increasing their frequency. This postulate especially applies to patient #006, who developed a subcutaneous and lung metastases at the end of the treatment, and concordantly, T cells targeting most private neoantigens increased in the Post 3 sample. It is more difficult to sustain this in the case of patient #032 since she remains free of detectable metastasis eight years after ending her treatment. So it would have to be assumed that she was harboring micrometastases that might have been controlled after vaccination.

Here we found that, even when the patient’s immune system recognizes multiple antigens not shared with their tumors, these responses do not impede the development of immune responses to TAA and neoantigens that might have therapeutic effects. Moreover, we observed a higher frequency of T cells targeting the tumor antigens compared to those targeting VACCIMEL off-target antigens, supporting the observation that the immune response against epitopes not expressed in the patient’s tumors was not detrimental to the relevant immune responses. In a previous study, James et al. (8) implemented an *in silico* approach to model the repertoire of antigens presented by allogeneic cancer cell lines, and to generate a quantitative comparison between potentially beneficial TAA and neoantigens versus distracting antigens offered by the hypothetical allogeneic vaccines. They found that alloantigens greatly outnumber shared TAA and neoantigens, suggesting that this fact may explain the generally limited clinical efficacy of allogeneic vaccines. However, this *in silico* study lacked the experimental validation of vaccinated patients’ immune responses to the distracting candidate epitopes. In our study, we were able to experimentally assess the immunogenicity of the best ranked candidate epitopes, validating that polymorphisms can be indeed antigenic, and comparing the magnitude of T cell immune responses targeting TAA, patient’s private neoantigens, and vaccine-derived epitopes, further demonstrating that VACCIMEL was capable to stimulate stronger responses towards the relevant antigens. Although our study does not disclose the role of non-tumor-specific antigens, here we provide the first experimental evidence of immunization towards a broad repertory of allo and cancer antigens in human allogeneic vaccination that paves the way to answer this question. In our view, the role of the alloantigens in whole-tumor cell vaccines should not be disregarded; perhaps they are acting as enhancers of the immune response. Accordingly, Li et al. (5) conducted a study in mice where they compared the immune response against two cellular melanoma vaccines: one autologous and the other transfected with an allogeneic major histocompatibility complex (MHC) molecule. Both treatments demonstrated a benefit, but the allo-immunotherapy produced a slightly better tumor growth control and a comparable therapeutic response in terms of mean survival time. The reported benefit was correlated with the number of activated DCs and T-cells in the tumor draining lymph nodes (TDLNs) of the treated animals. Additionally, the allo-immunotherapy was capable of stimulating T cells directed to the tumor antigens Trp1, Trp2, gp100, and tyrosinase.

This research was also motivated by the need to determine the amount of neoantigens offered by VACCIMEL. Our study was limited by the lack of access to patient samples, so we only studied shared neoantigens of patient #032, who had the largest number and best ranked neoepitope candidates. Interestingly, this patient presented an extremely high TMB, probably increasing the chances of sharing such neoantigens. We found T cells targeting shared neoepitopes of this patient, but surprisingly, these immune cells had a lower frequency in comparison to the ones targeting private neoantigens and TAA. To overcome the limitations of our study, we investigated melanoma tumors from another cohort (15, 36), and concordantly we found a correlation between the patient’s TMB and the amount of predicted shared neoepitopes. This analysis also revealed that most melanoma tumors share somatic mutations with the vaccine that may generate TSA, however, there is little evidence of their immunogenicity. Therefore, we conclude that VACCIMEL might provide shared neoantigens, but these would not be the main drivers of the therapeutic antitumor immune responses.

In the present study, we implemented a computational pipeline that allowed us to identify alloantigens and neoantigens in cellular vaccines, such as VACCIMEL. This approach involved the usage of MuTect2, a software designed to identify somatic mutations in a novel way, by pairing exomes from different sources. Further, we took advantage of bioinformatic tools designed to predict neoepitopes, allowing us to rank epitope candidates of a different nature, such as alloantigens. By inspecting the characteristics of all the epitopes recognized following vaccination, we found an association between their mRNA abundance in VACCIMEL cells and the magnitude of the T cell responses directed to them. This observation is in line with previous studies that highlight the role of antigen expression in immunogenicity (46–49). This factor increases the number of VACCIMEL antigens loaded onto the patient’s APCs HLA molecules, contributing to a higher overall T cell avidity (50). However, it is important to mention that the metrics used in this study (AUC to evaluate the presence of immune response and Pearson’s correlation coefficient to evaluate its intensity) demonstrated a quite limited performance of all the explored peptide features. This can be attributed to the fact that the same peptide triggered variable immune responses at different time points and in different patients, as also observed by Borch et al. (36). Therefore, it is possible that many of the peptides identified here as negatives actually have properties that allow them to be processed, presented, and even recognized by T cells, as described by the calculated features (36). However, the generation of specific TCRs with rearrangements capable of recognizing them, or the general immune state of the patient, may hinder the triggering of cellular immune responses. Also, it is not clear whether the patient’s fluctuation of peptide-specific T cells is a true reflection of its immune state evolution, or if this observation is associated with the fact that testing was performed with PBMC samples that might have a very low frequency of VACCIMEL-specific and tumor-specific T cell clones. We have previously performed TCRb sequencing of TILs of tumors of patients #006 and #045, and observed that more than 50% of TILs are not present in the blood (26, 51), suggesting that tumor-specific T cells might be enriched in the tissues, but here we could not test post-vaccination TILs due to lack of fresh tumor samples. In either case, a unique ELISpot negative result appears to be insufficient to annotate a peptide as non-immunogenic.

In a context in which personalized cancer vaccines are flourishing thanks to the development of improved vaccination platforms such as mRNA and neoantigen detection algorithms, it is reasonable to wonder if allogeneic whole-tumor vaccines are still valid. Allogeneic whole-tumor vaccines express common, shared, highly immunogenic non-mutated TAA, which are important targets of the immune response elicited by vaccination, as we have previously demonstrated for VACCIMEL (21). Also, they present the benefit of being an off-the-shelf therapeutic strategy that can be quickly administered after the malignancy is diagnosed, and that can be prescribed to cancer patients without studying their tumor samples. Additionally, these vaccines carry the highly immunogenic HLA; although here we have not studied HLA-specific T cell responses, we predicted several epitope candidates derived from these molecules and antibody production against VACCIMEL’s allogeneic HLAs has been previously shown (52). These anti-HLA antibodies might opsonize the vaccine cells in successive immunizations and contribute to enhance the capture and processing by APC at the vaccination site. Whole-tumor cell vaccinations also offer entire antigens in comparison to peptidic fragments, which are capable of stimulating both CD4 and CD8 T cell, as well as B cell responses, providing a broad effector repertoire. Nevertheless, it is important to review the possible causes of the limited effectiveness of whole-tumor cell vaccines observed in multiple clinical trials (7).

We believe that VACCIMEL has extended the DMFS in treated melanoma patients (17) due to the powerful immunostimulation generated by the Th1-polarizing BCG, the high and locally sustained doses of GM-CSF, the selection of highly transformed melanoma cell lines expressing most TAA, the use of irradiation as a method to generate apoptosis and necrosis of the tumor cells serving as a sign of damage, and possibly due to the contribution of highly immunogenic alloantigens such as HLA. It can be argued that personalized vaccines immunize against private neoepitopes that, as we have shown here, are not included in allogeneic cell lines. Even when VACCIMEL immunotherapy stimulated immune responses to private neoantigens, it cannot be ruled out that a vaccination against other predicted neoepitopes could have improved the antitumor effect. Therefore, the two vaccination approaches appear to be complementary, and it is likely that their combination may lead to a synergistic effect capable of enhancing the overall response in cancer patients. Furthermore, other immunotherapeutic strategies, such as the administration of anti-PD-1 monoclonal antibodies relieve tumor-specific T cells through the PD-1/PDL-1 axis can contribute to control cancer cells (53). Recently, we have reported that 5/5 vaccinated patients who progressed even years after ending VACCIMEL treatment presented complete responses to anti-PD-1 treatment (25). We hypothesize that the diverse VACCIMEL-induced immune stimulation could become memory cells and contribute to control metastasis that reappeared even years following vaccination, after their recall from secondary lymphoid organs and reinvigoration with anti-PD1 treatment.

## 4 Conclusion

Allogeneic whole-tumor cell vaccination with VACCIMEL stimulated a broad immune response against tumor-associated antigens, neoantigens, and alloantigens. Our results have shown that the immunogenicity of off-target antigens did not prevent immune responses against TAA and TSA, which are the most likely target antigens for therapeutic effect of antitumor vaccination.

## 5 Materials and methods

### 5.1 Vaccine antigen prediction

#### 5.1.1 Whole-exome sequencing

To perform WES, total DNA from each VACCIMEL cell line was extracted. VACCIMEL cell lines were developed as previously described (18). In addition, DNA from PBMC samples of selected vaccinated patients were obtained to use as germline DNA. Using Covaris technology, the genomic DNA samples were randomly fragmented in sizes between 200 and 300 bp. The resultant fragments’ ends were then ligated to adapters. To enrich the extracted DNA, it was purified, amplified using ligation-mediated PCR (LM-PCR), and hybridized to the exome array Agilent SureSelect Human All Exon V5 (50M) Library. Non-hybridized fragments were removed. In total, 50.39 megabase target regions were captured. To calculate the enrichment, quantitative PCR and the Agilent 2100 Bioanalyzer were applied to the captured LM-PCR products. After loading each eligible captured library onto an Illumina Hiseq 4000 PE100 platform, high-throughput sequencing was performed. Raw image files were processed by Illumina base calling software with default parameters and the sequence data of each sample was generated as paired-end reads, and stored in FASTQ format (BGI Americas).

The sequencing raw data was processed according to GATK best practices (54). Briefly, the FASTQ files’ quality was evaluated with FastQC version 0.11.9. To handle low-quality reads, FASTQ files were processed with trim-galore version 0.6.10. Burrows-Wheeler Aligner version 0.7.17 mem algorithm (55) was used to align the trimmed reads to the human reference genome version GRCh38. Picard-tools MarkDuplicates version 2.26.2 was applied to tag duplicated reads associated with technical artifacts, and the quality scores were recalibrated with GATK version 4.2.6.1. Coding regions were extracted from BAM files using bedtools intersect (56). The average coverage in these regions was 75.

#### 5.1.2 Variant calling

We aimed to detect the genomic variants in VACCIMEL cell lines that are not present in the vaccinated patients’ genomes, and that therefore can be the source of allo or cancer antigens. For this, MuTect2 from GATK version 4.2.6.1 was applied to the bam files from each vaccine cell line paired to the bam files from the germline samples of the patients. Variants called were filtered with FilterMutectCalls from GATK version 4.2.6.1, merged with bcftools version 1.6 (57), and annotated using Ensembl Variant Effect Predictor (VEP) v105 (58).

Also, VACCIMEL’s somatic mutations were identified with MuTect2 from GATK version 4.2.6.1 in tumor only mode, using the Genome Aggregation Database (gnomAD) v4.1.0 based in exome and genome samples (59) as a germline resource. Mutational signatures of VACCIMEL cell lines were determined with SigProfilerAssignment (60).

To identify the proportion of cancer-related VACCIMEL variants that according to the patient’s germinal background might generate neoantigens, VACCIMEL’s somatic mutations were intersected with VACCIMEL variants using bcftools (56).

#### 5.1.3 RNAseq

RNASeq of VACCIMEL’s cell lines was performed for VACCIMEL cell lines using the Illumina HiSeq TM 2000 sequencing technology (BGI Americas), as previously described (18). Briefly, mRNA is enriched and fragmented. cDNA is synthesized, purified, ligated to adaptors, and amplified by PCR. Agilent 2100 Bioanalyzer and ABI StepOnePlus Real-Time PCR System are used to qualify and quantify the sample library, and the library products are sequenced by Illumina HiSeq TM 2000. Raw reads are subjected to quality control (QC) and filtered into clean reads.

Transcript abundance was quantified with Kallisto version 0.46.0 (61). Genes were tagged with Hugo Symbol (gencode v25) and abundance was expressed in transcripts per million (TPM). To evaluate the gene expression of the vaccine as a whole, the gene expression of the four cell lines was summed and the values were rescaled to sum one million so that they are expressed in TPM. Besides, the expression of the identified mutated transcripts was verified. For this, RNA reads were mapped to the human reference genome using STAR version 2.7.10 (62) and the variant allele frequency (VAF) was calculated as the proportion of mutated reads among total reads covering the mutated position using Samtools mpileup version 1.13 (57).

#### 5.1.4 Patient’s HLA typing

Patients’ HLA class I and II haplotypes were determined on PBMC isolated from the vaccinated patients by high-resolution genotyping (Scisco Genetics) (63). Further, we verified the HLA genotype of patient #045 using the WES of healthy tissue with Optitype version 1.2 (33).

#### 5.1.5 Prediction and prioritization of T cell epitopes derived from variants

T cell epitope candidates deriving from the identified VACCIMEL exomic variants were predicted. Variants were included if at least one out of the four vaccine cell lines passed the MuTect2 quality filters (PASS). Then, variants were annotated with VEP v105 and only the ones that were located in coding regions and that generated non-synonymous alterations were considered. The peptide extraction was performed using MuPeXi 1.2.0 (37) with VEP 95.1 (58) and NetMHCpan 4.0 (64). Further, the likelihood of presentation of the peptide on the HLA was re-calculated with NetMHCpan 4.1 (28), and the stability of the pHLA complex was predicted with NetMHCstabpan 1.0 (65).

The epitope candidates were selected for experimental evaluation according to the following criteria: at least one cell line had the variant with a variant allele frequency higher than %10, the gene was expressed in the vaccine cell lines (TPM > 0), each variant transcript was detected when the mutated position had at least 10 reads of RNAseq coverage, the mutant peptide had a predicted strong binding to HLA (%Rank_EL < 0.5), the wild type peptide was predicted to not bind to HLA (%Rank_EL ≥ 2), and the mutated peptide had a predicted stable binding to HLA (%Rank_Stab < 2). Peptides predicted to bind to HLA C were excluded because NetMHCstabpan was not trained with data from this class. From the candidates filtered according to these criteria, the final peptide selection was manually curated optimizing for dispersion in stability and expression values, and excluding similar peptides originating from the same variant.

#### 5.1.6 Prediction of T cell epitopes derived from allogeneic HLA

To identify T cell epitope candidates deriving from VACCIMEL’s allogeneic HLA molecules, the HLA haplotype of the component melanoma cell lines was determined with high resolution using Optitype version 1.2 (33) using the WES data. The results were concordant with the typing performed with serological methods (35). The complete HLA A, B and C protein sequences corresponding to the identified alleles were retrieved from the IPD-IMGT/HLA Database (34). Then, we identified mismatches with the patient’s corresponding HLA molecules and peptides containing the mismatches were used as inputs to NetMHCpan 4.1 (28) and NetMHCIIpan 4.3 (30). Only predicted strong binders (%Rank_EL < 0.5 for NetMHCpan-4.1, and %Rank_EL < 1.0 for NetMHCIIpan-4.3) were considered for this analysis.

### 5.2 Prediction of patient’s tumor neoantigens shared with VACCIMEL

The patient’s tumor’s neoantigen prediction from somatic mutations was performed as described in (22). Briefly, patient’s samples from tumor and healthy tissues were obtained as described in (18). Paired WES was performed like in (21), and somatic variants were identified using MuTect2 (66) following GATK best practices. MuPeXi (37) was used to obtain the neopeptide sequences between 8 and 11 amino acids length, and candidate neoepitopes were chosen based on predicted antigen presentation on the patient’s HLA using NetMHCpan 4.0 (59) (%Rank_EL < 2). The candidates were intersected with VACCIMEL data to search for shared neoepitope candidates. Finally, a set of candidates was manually curated to evaluate the immune response. Additionally, some peptides previously tested and published in (22) were included in the dataset analyzed here.

#### 5.2.1 Tumor mutational burden calculation

The patient’s TMB was calculated from the exomic variants identified with MuTect2 and annotated with VEP v105, using the Institut Curie TMB analysis tool (67). Non-coding and synonymous variants were filtered. A human exome of 50.39mb was considered for this analysis.

### 5.3 Immunogenicity assessment

To assess the epitope candidates’ immunogenicity, patients’ PBMC were obtained during the treatment with VACCIMEL (18). Using these samples, synthetic peptides (Royo Biotech) were tested *in vitro* in IFNγ ELISpot assays (BD human IFN-γ ELISPOT Set) at 5ug/ml in an effector-target relation of 1:10, following the protocol described in (21). Peptides were tested in pools and positive pools were studied individually. All assays were performed with post 3 samples (collected after 2 years of the treatment consisting of 13 vaccinations), except for patient #006 in which, due to sample scarcity, the effector cells used for the individual assessment of peptides were a mixture of post 1 (after 6 months of treatment and 5 vaccinations) and post 3 samples. Plates were read with an AID EliSpot Reader System.

The immune response magnitude or intensity corresponds to the average number of spots observed on peptide-stimulated effector cells replicates minus the number of unspecific spots observed in unstimulated effector cells (cultured with non-pulsed APC), relative to 100.000 effector cells. Peptides were considered immunogenic (positive) according to the distribution assumption free resampling (DFR) test (68) when triplicates were available and reliable, and empirical criteria were applied otherwise.

### 5.4 Peptide feature calculation

The mutation consequence and the number of mismatches were obtained from the MuPeXI output (38).

Gene expression in healthy melanocytes was obtained from the Human Protein Atlas (69). Additionally, peptide expression levels were obtained from the TCGA skin cutaneous melanoma (SKCM) dataset using pepX (70). From these data, we calculated the fold change of expression between healthy melanocytes and tumor samples as log_2_(Tumor_exp_/Melanocyte_exp_), where Tumor_exp_ was derived from VACCIMEL RNAseq and TCGA SKCM datasets. Additionally, the expression of the mutated transcripts was calculated as VACCIMEL_exp_ x RNA_VAF_.

Proteasome and immunoproteasome cleavage predictions were calculated with ProteaSMM (71). TAP transport efficiency was predicted with TAP-SMM (72).

The likelihood of peptide presentation on the patient’s HLA class I molecules was predicted with NetMHCpan 4.1 (28). Predictions of the NetMHCpan method trained with Binding Affinity (BA) and Mass-Spectrometry Eluted Ligands (EL) peptides were retrieved. Predictions of antigen processing, antigen presentation, and the presentation score were calculated with MHCflurry 2.0 (73). Also, presentation on the patient’s HLA was predicted with MixMHCpred 2.2 (74).

The difference between the binding of the mutant and wild-type peptides, named the Differential agretopicity index (DAI) was calculated as Mutant %Rank_EL/Normal %Rank_EL, according to (75).

The HLA promiscuity of the peptides defined as their presentation on more than one HLA was evaluated as the number of alleles that the peptide is predicted to bind with weak and strong affinity. Additionally, an aggregated score of peptide presentation on all the patient’s HLA class I alleles, named the Patient Harmonic-mean Best Rank (PHBR), was calculated similarly to the scoring described in (76).

The stability of the pHLA complex was predicted with NetMHCstabpan 1.0 (65).

Predictions of antigen presentation by integrating HLA binding and gene expression values derived from RNASeq were calculated with NetMHCpanExp 1.0 (46). The expression values used to perform the predictions were derived from the RNAseq of VACCIMEL cell lines and the TCGA skin cutaneous melanoma (SKCM) dataset.

Since anchor residues contact HLA, TCRs are more likely to interact with non-anchor residues. These amino acids were derived from the predicted HLA binding Icore obtained NetMHCpan-4.1, by selecting the fourth to the penultimate residues, as in (36). Sequence similarity between mutant and wild-type peptides was calculated over peptides and peptides without anchor positions using the Alignment-free Kernel Distance (77).

Dissimilarity and foreignness scores were measured with antigen.garnish (78).

T cell recognition was predicted with PRIME 2.0 (74). T cell propensity was predicted with (79). The immunogenicity of the peptides was predicted with ICERFIRE 1.0 (80) and with the IMPROVE simple model (36).

The physicochemical properties of the peptides were calculated with the BioPython ProteinAnalysis module (81).

In the cases that the computational methods required the patient’s HLA information, predictions were performed for all 6 HLA class I molecules, and the best value was conserved.

### 5.5 Statistical analysis

All statistical analyses were performed using Python 3.8.10. Wilcoxon rank-sum test or Wilcoxon-Mann-Whitney U test was calculated with SciPy (82) and it was used to analyze the differences between immunogenic and non-immunogenic peptides according to calculated features, and between immunogenic peptides from different sources. Statistical significance was assessed as: * p < 0.05; ** p < 0.01; *** p < 0.001; **** p < 0.0001; ns, non significant. Pearson’s correlation coefficients used to measure the correlation between features and IFNγ ELISpots were also calculated with SciPy (82). Predictive performance was evaluated in terms of AUC, which was calculated using the scikit-learn package (83).

## Data Availability

WES and RNAseq data from the four human melanoma cell lines that compose VACCIMEL are not publicly available since they are protected by a US patent (12/450,721/US2010183683). Requests to access the datasets should be directed to barrio.marcela@gmail.com.
