# Supplementary material for: VACCIMEL, an allogeneic melanoma vaccine, efficiently triggers T cell immune responses against neoantigens and alloantigens, as well as against tumor-associated antigens
Source: Front Immunol. 2025 Jan 7;15:1496204. doi: 10.3389/fimmu.2024.1496204 (PMC11747570; doi:10.3389/fimmu.2024.1496204)
Supplement: Supplementary file 1 [file DataSheet1.docx]

***Supplementary Material***

# Supplementary Figures


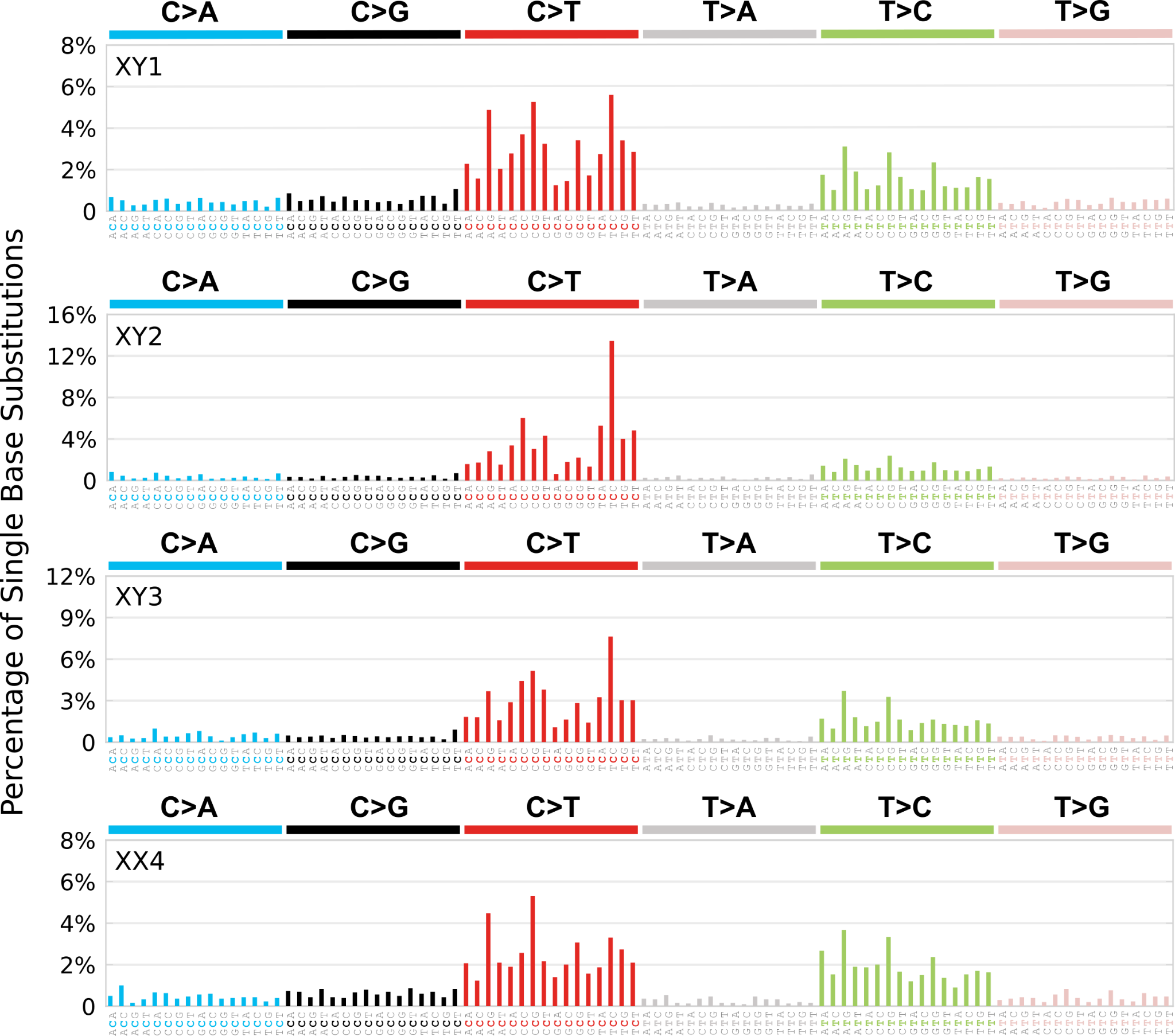


Supplementary Figure 1: Single base substitution mutational profiles of VACCIMEL cell lines estimated with SigProfiler (1).


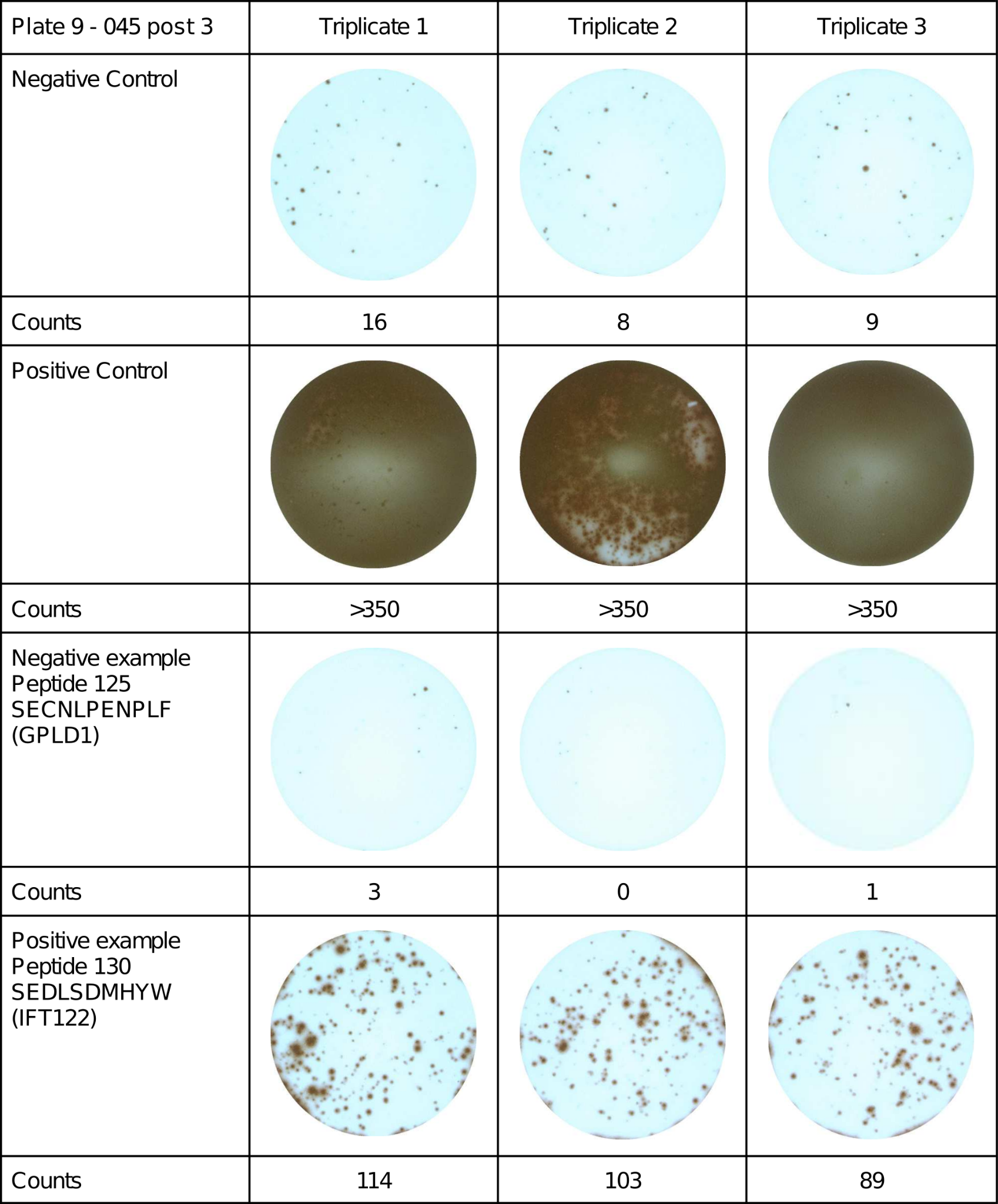
Supplementary Figure 2: ELISpot visualization example.


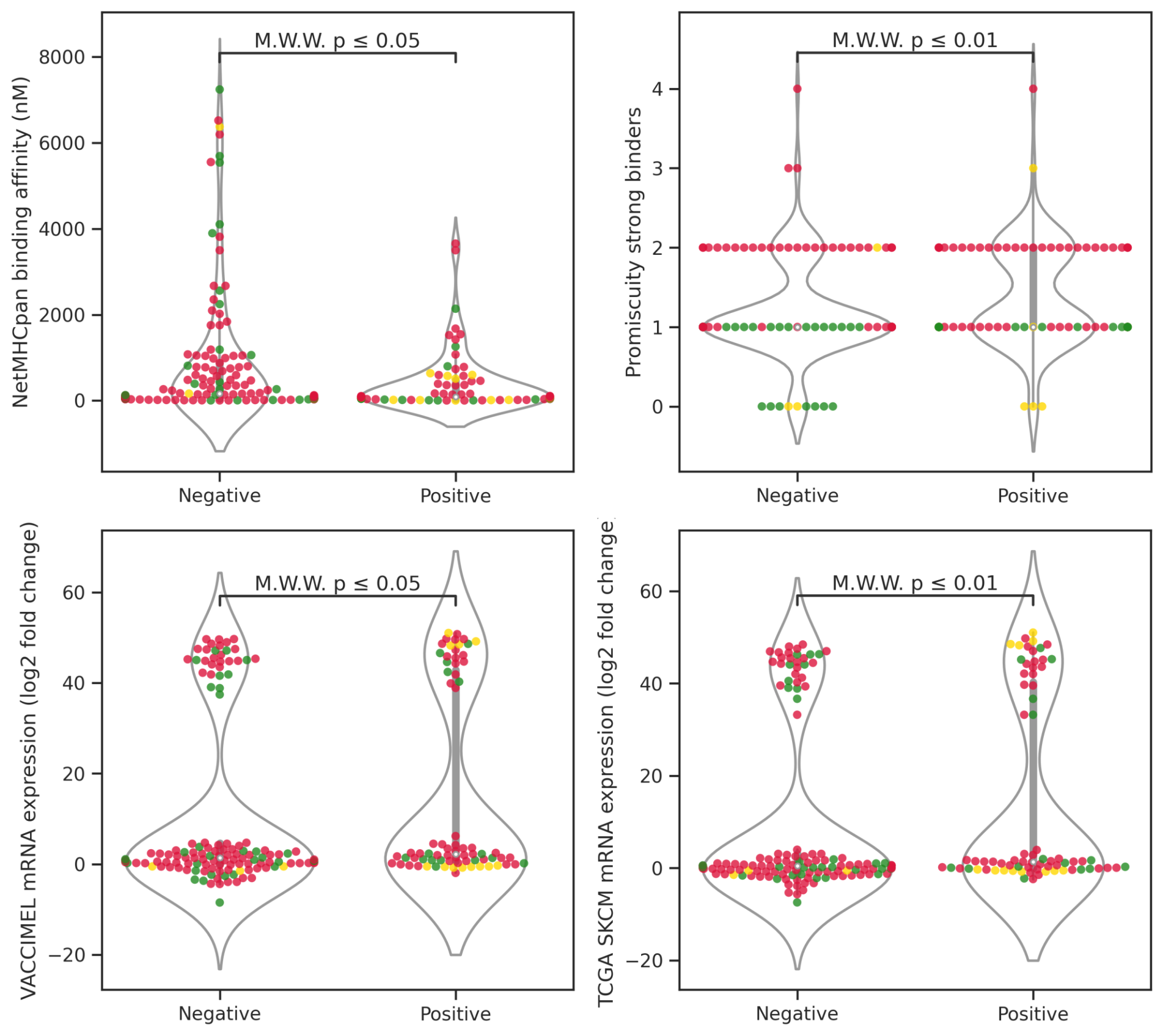


Supplementary Figure 3: Features of VACCIMEL peptides that are different in epitopes and non-epitopes. Only the features that provided a statistically significant difference are reported here. Immunogenic peptides are colored according to their source. Red: peptides exclusive from the vaccine or category V. Green: shared neopeptides expressed both in the tumor and the vaccine or category TV. Yellow: TAA or category TVG (2). The x-axes represent the result of IFNγ ELISpot and the y-axes the different features.


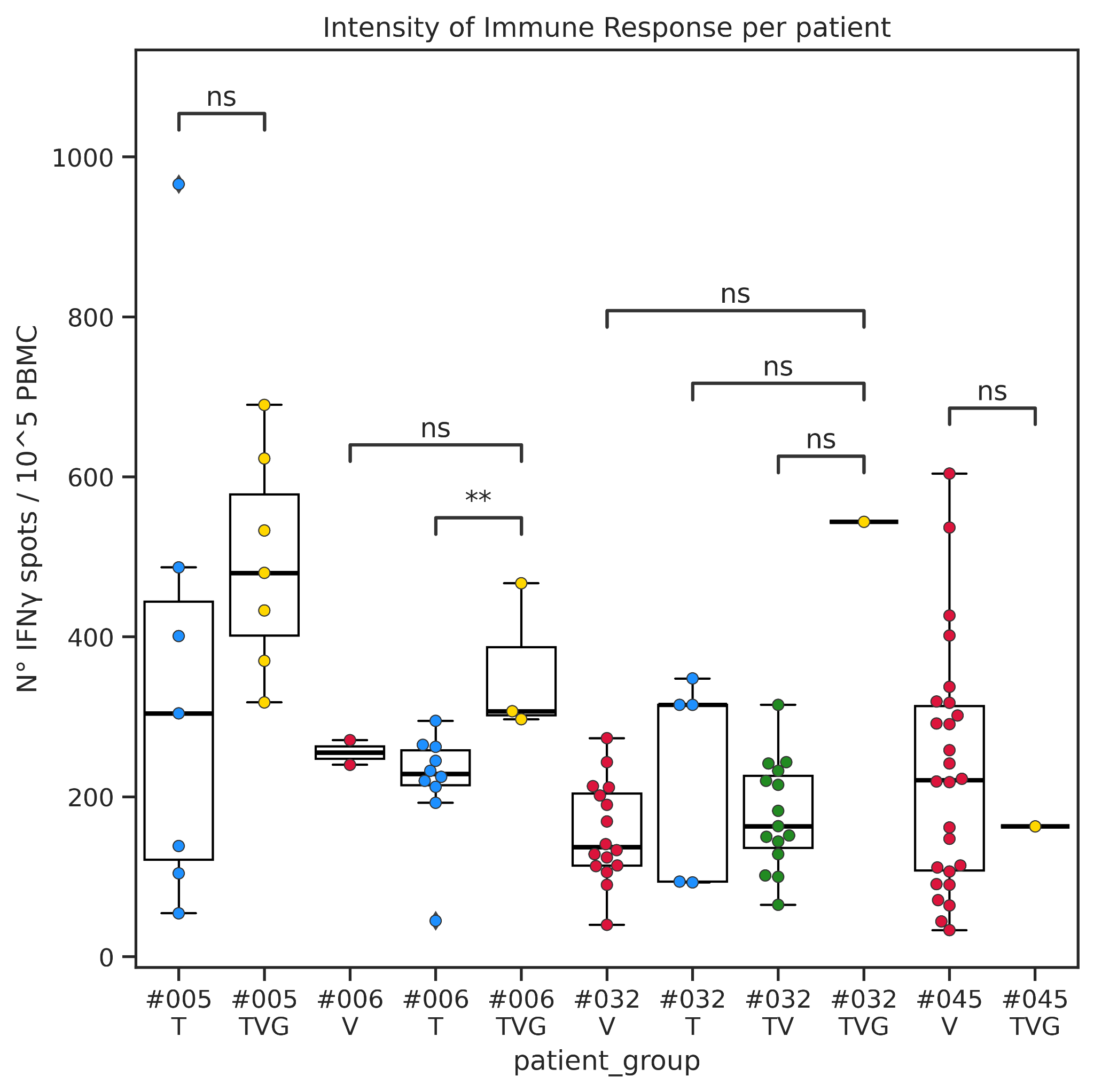


Supplementary Figure 4: Intensity of T cell responses evaluated as reactive T cell frequency in ELISpot assay per antigen category and patient. V: peptides exclusive from the vaccine. TV: shared neopeptides expressed both in the tumor and the vaccine. T: private neopeptides previously tested in (3). TVG: TAA expressed both in the vaccine, tumor and germinal, previously tested in (2). The y-axis represents the intensity of IFNγ ELISpot in terms of the number of spots observed per 100000 effector cells minus background spots. The statistics were calculated using the Mann-Whitney-Wilcoxon test. **p < 0.01; ns = non significant.


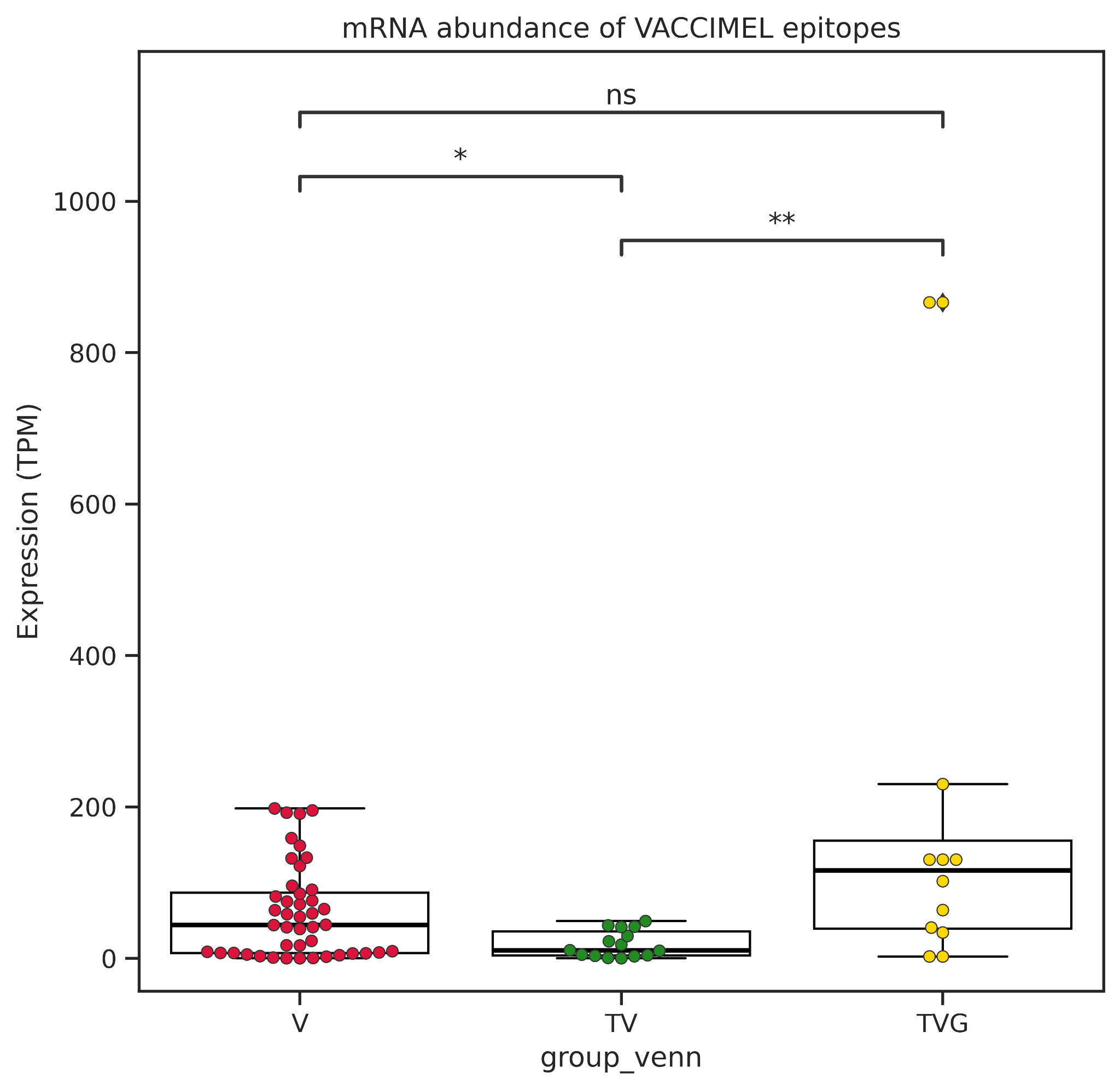


Supplementary Figure 5: Gene expression in TPM of VACCIMEL epitopes per category. * p < 0.05; **p < 0.01; ns = non significant.

#

# Supplementary Tables

Supplementary Table 1: Analysis of somatic mutations in VACCIMEL cell lines. Variants were identified using MuTect2 in tumor-only mode since germline samples were not available. The contribution of known mutational signatures to VACCIMEL’s mutational profiles was estimated with SigProfiler (1). SBS1 is an endogenous mutational process initiated by spontaneous or enzymatic deamination of 5-methylcytosine to thymine which generates G:T mismatches in double-stranded DNA. Failure to detect and remove these mismatches prior to DNA replication results in fixation of the T substitution for C. SBS5 is increased in bladder cancer samples with ERCC2 mutations and in many cancer types due to tobacco smoking. SBS7a/SBS7b/SBS7c/SBS7d are found in cancers of the skin from sun-exposed areas and are thus likely to be due to exposure to ultraviolet light. SBS26 proposed etiology is defective DNA mismatch repair. SBS54 etiology is a possible sequencing artifact or contamination with germline variants (4).

|  | **No. Somatic mutations** | **SBS1** | **SBS5** | **SBS7a** | **SBS7b** | **SBS26** | **SBS54** |
| --- | --- | --- | --- | --- | --- | --- | --- |
| **XY1** | 11702 | 14.1% | 58.4% | 5.72% | 13.37% | 0% | 8.4% |
| **XY2** | 4639 | 7.89% | 34.23% | 22.52% | 24.15% | 11.22% | 0% |
| **XY3** | 4614 | 11.03% | 52% | 8.98% | 17.14% | 0% | 10.86% |
| **XX4** | 3587 | 12.85% | 68.78% | 0% | 8.25% | 0% | 10.12% |

Supplementary Table 2: Tumor Mutational Burden (TMB) expressed as mutations per megabase in vaccinated patients. The TMB level is categorized according to (5).

|  | **TMB** | **TMB level** |
| --- | --- | --- |
| #005 | 21.5 | high |
| #006 | 17.6 | intermediate |
| #032 | 526.3 | high |
| #045 | 38.8 | high |

Supplementary Table 3: HLA typing of vaccinated patients

| Patient | A | B | C | DPA1 | DPB1 | DQA1 | DQB1 | DRB1 | DRB345 |
| --- | --- | --- | --- | --- | --- | --- | --- | --- | --- |
| #005 | 02:01:01 | 07:02:01 | 07:01:01 | 01:03:01 | 04:01:01 | 01:02:01 | 03:01:01 | 11:04:01 | DRB3*02:02:01 |
|  | 03:01:01 | 18:01:01 | 07:02:01 | 01:03:01 | 04:01:01 | 05:05:01 | 06:02:01 | 15:01:01 | DRB5*01:01:01 |
| #006 | 11:01:01 | 51:01:01 | 06:02:01 | 01:03:01 | 02:01:02 | 02:01 | 03:02:01 | 04:01:01 | DRB4*01:01:01 |
|  | 25:01:01 | 57:01:01 | 15:02:01 | 01:03:01 | 04:01:01 | 03:01:01 | 03:03:02 | 07:01:01 | DRB4*01:03:01 |
| #032 | 01:01:01 | 51:01:01 | 05:01:01 | 01:03:01 | 01:01:02 | 01:02:01 | 03:03:02 | 07:01:01 | DRB3*03:01:01 |
|  | 31:01:02 | 57:01:01 | 07:01:01 | 02:01:01 | 04:01:01 | 02:01:01 | 06:09:01 | 13:02:01 | DRB4*01:03:01:02 |
| #045 | 29:02:01 | 44:03:01 | 16:01:01 | 01:03:01 | 04:01:01 | 02:01:01 | 02:02:01 | 07:01:01 | DRB3*02:02:01 |
|  | 29:02:01 | 44:03:01 | 16:01:01 | 02:01:01 | 11:01:01 | 05:05:01 | 03:01:01 | 11:01:01 | DRB4*01:01:01:01 |

Supplementary Table 4: Tested peptides, immune assessment, calculated features, and additional information associated with each peptide. * Average number of spots observed on peptide-stimulated effector cells replicates minus the number of unspecific spots observed in unstimulated effector cells (cultured with non-pulsed APC), relative to 100.000 effector cells. † Immune response was determined by DFR test, as described under methods. 1 = Immunogenic; 0 = Non immunogenic; TSG = Tumor Suppressor Gene; M = Missense variant; F = Frameshift variant; D = Inframe deletion; N = No variant.

*This table was supplied as an excel file.*

Supplementary Table 5: Expression level of HLA molecules in VACCIMEL cell lines.

|  | **Expression (TPM)** | | |
| --- | --- | --- | --- |
|  | **HLA-A** | **HLA-B** | **HLA-C** |
| MEL-XY1 | 341.5 | 162.9 | 368.7 |
| MEL-XY2 | 291.5 | 263.4 | 193.7 |
| MEL-XY3 | 398.27 | 69.4 | 282.47 |
| MEL-XX4 | 577.25 | 707.05 | 1227.96 |

Supplementary Table 6: VACCIMEL epitope candidates [predicted HLA strong binders according to NetMHCpan 4.0 (6) or NetMHCIIpan 4.3 (7)] derived from polymorphisms in the HLA A, B and C loci.

|  | **Patient** | | | |
| --- | --- | --- | --- | --- |
|  | **#005** | **#006** | **#032** | **#045** |
| No. VACCIMEL HLA class I candidate epitopes presented in the patient’s HLA class I molecules | 23 | 53 | 64 | 58 |
| No. VACCIMEL HLA class I candidate epitopes presented in the patient’s HLA class II molecules | 79 | 63 | 81 | 168 |

Supplementary Table 7: Performance metrics of peptide features.

*This table was supplied as an excel file.*

Supplementary Table 8: Mutated genes originating the shared predicted neoepitopes between VACCIMEL and the patient’s tumors. The function annotation was obtained from GeneCards (8).

| **Gene** | **Description** | **No. of patients** | **Function** |
| --- | --- | --- | --- |
| APBB1IP | Amyloid Beta Precursor Protein Binding Family B Member 1 Interacting Protein | 1 | It appears to function in the signal transduction from Ras activation to actin cytoskeletal remodeling. Suppresses insulin-induced promoter activities through AP1 and SRE. Mediates Rap1-induced adhesion. |
| BRAF | B-Raf Proto-Oncogene, Serine/Threonine Kinase | 9 | Protein kinase involved in the transduction of mitogenic signals from the cell membrane to the nucleus. |
| MST1/HGFL | Macrophage Stimulating 1 | 1 | Macrophage stimulating protein 1, paralog to HGF. |
| RP1L1 | Retinitis pigmentosa 1-like 1 | 1 | Involved in the differentiation of photoreceptor cells. |
| STRA6 | Signaling Receptor and Transporter of Retinol STRA6 | 1 | Retinol Transporter. |
| TDG | Thymine DNA Glycosylase | 1 | DNA glycosylase that plays a key role in active DNA demethylation. |
| TRGC1 | T Cell Receptor Gamma Constant 1 | 1 | Constant region of T cell receptor gamma chain that participates in antigen recognition. |
| ZFPM2 | Zinc Finger Protein, FOG Family Member | 1 | Transcription regulation involved in heart morphogenesis. |
| ZNF300 | Kruppel-like Zinc Finger Protein 300 | 1 | Transcriptional repressor. |
| ZNF41 | Zinc Finger Protein 41 | 1 | Transcriptional regulator. |

#

# References

1. Díaz-Gay M, Vangara R, Barnes M, Wang X, Islam SMA, Vermes I, et al. Assigning mutational signatures to individual samples and individual somatic mutations with SigProfilerAssignment. Bioinformatics. 2023 Dec 1;39(12):btad756. Available from: <http://dx.doi.org/10.1093/bioinformatics/btad756>

2. Podaza E, Carri I, Aris M, von Euw E, Bravo AI, Blanco P, et al. Evaluation of T-Cell Responses Against Shared Melanoma Associated Antigens and Predicted Neoantigens in Cutaneous Melanoma Patients Treated With the CSF-470 Allogeneic Cell Vaccine Plus BCG and GM-CSF. Front Immunol. 2020 Jun 5;11:1147. Available from: <http://dx.doi.org/10.3389/fimmu.2020.01147>

3. Carri I, Schwab E, Podaza E, Garcia Alvarez HM, Mordoh J, Nielsen M, et al. Beyond MHC binding: immunogenicity prediction tools to refine neoantigen selection in cancer patients. Explor Immunol. 2023 Apr 25;3(2):82–103. Available from: <https://doi.org/10.37349/ei.2023.00091>

4. Alexandrov LB, Nik-Zainal S, Wedge DC, Aparicio SAJR, Behjati S, Biankin AV, et al. Signatures of mutational processes in human cancer. Nature. 2013 Aug 22;500(7463):415–21. Available from: <http://dx.doi.org/10.1038/nature12477>

5. Chalmers ZR, Connelly CF, Fabrizio D, Gay L, Ali SM, Ennis R, et al. Analysis of 100,000 human cancer genomes reveals the landscape of tumor mutational burden. Genome Med. 2017 Apr 19;9(1):34. Available from: <http://dx.doi.org/10.1186/s13073-017-0424-2>

6. Jurtz V, Paul S, Andreatta M, Marcatili P, Peters B, Nielsen M. NetMHCpan-4.0: Improved Peptide-MHC Class I Interaction Predictions Integrating Eluted Ligand and Peptide Binding Affinity Data. J Immunol. 2017 Nov 1;199(9):3360–8. Available from: <http://dx.doi.org/10.4049/jimmunol.1700893>

7. Nilsson JB, Kaabinejadian S, Yari H, Kester MGD, van Balen P, Hildebrand WH, et al. Accurate prediction of HLA class II antigen presentation across all loci using tailored data acquisition and refined machine learning. Sci Adv. 2023 Nov 24;9(47):eadj6367. Available from: <http://dx.doi.org/10.1126/sciadv.adj6367>

8. Stelzer G, Rosen N, Plaschkes I, Zimmerman S, Twik M, Fishilevich S, et al. The GeneCards Suite: From Gene Data Mining to Disease Genome Sequence Analyses. Curr Protoc Bioinformatics. 2016 Jun 20; 54(1), 1-30. Available from: <http://dx.doi.org/10.1002/cpbi.5>
